# Vasectomy: A simple snip?

**DOI:** 10.4103/0970-1591.30254

**Published:** 2007

**Authors:** Nivedita Bhatta Dhar, J. Stephen Jones

**Affiliations:** Glickman Urological Institute, The Cleveland Clinic Foundation, Cleveland, Ohio, USA

**Keywords:** Azoospermia, semen analysis, vasectomy

## Abstract

Vasectomy is one of the most common forms of permanent sterilization methods currently in use and has a failure rate of <1% in most reported series. Since failure of vasectomy may result in pregnancy, adequate counseling is essential. Couples are advised that an analysis of a semen specimen after vasectomy is required to confirm success before the use of alternative contraception is abandoned. However, measuring the success of vasectomy is complicated by a lack of consistency with regards to both the number and timing of tests and the end points accepted.

**Materials and Methods:**

A Medline search was used to identify manuscripts dealing with vasectomy, with specific attempts to identify protocols designed to confirm sterility.

**Results and Conclusion:**

Vasectomy is one of the most reliable permanent methods of contraception. However, despite its popularity, certain issues pertaining to the procedure remain unresolved. Debate continues over the relative merits of the various techniques of isolating and sealing the vasal ends. Postoperative complication rates remain minimal regardless of the technique used, and no single strategy attempting to maximize patient compliance with postoperative semen analysis has enjoyed unmitigated success. Long-term consequences, other than regret, are rare.

Vasectomy is a safe and reliable means of contraception that is used by 42 million couples worldwide.[1] As failure of vasectomy may result in pregnancy, adequate counseling is essential. Couples are advised that analysis of a semen specimen after vasectomy (SSAV) is required to confirm success before the use of alternative contraception is abandoned. This review considers the literature supporting the recommended approaches and the issues involved in determining the success of vasectomy.

The timing and the number of specimens required to confirm success remains controversial because of variable clearance times of residual sperm from the ampul1a of the vas deferens and seminal vesicles. There exist no standardized guidelines in the follow-up of these patients to assess the efficacy of the vasectomy.[1] In addition recent reports indicate poor compliance in following instructions for determining sterility in this group of patients with most of the protocols in use.[2–4] Measuring the success of vasectomy is complicated by a lack of consistency with regards to both the number and timing of tests and the end points accepted. Classically, the absence of sperm in the SSAV was required to establish the success of the vasectomy. However, other investigators have suggested that achieving azoospermia after vasectomy is not an absolute requirement.[5] It was proposed that a man can be considered infertile as long as the spermatozoa present in the SSAVs are not motile.[6] However, testing for loss of motility relies on the patient delivering the semen sample within a short time of producing it which is not always feasible.

## MATERIALS AND METHODS

A Medline search was used to identify manuscripts dealing with vasectomy, with specific attempts to identify protocols designed to confirm sterility.

### Compliance

Postvasectomy semen analysis is critical to establish the success of vasectomy as a sterilization procedure. Studies have shown that up to 90% of urologists require two semen samples routinely and that up to 95% request further semen samples if nonmotile sperm were present.[1] However, many patients fail to follow postoperative instructions to obtain semen analysis and in a recent study, only 21% of patients followed recommendations to have two consecutive azoospermic readings despite aggressive counseling and education techniques.[4]

The reasons for poor compliance are unknown and therefore adequate prevasectomy counseling is essential.[4] Smucker *et al* surveyed 141 postoperative vasectomy patients because of a concern regarding their poor response rate for postoperative semen analysis, where 29% returned one specimen, 26% returned two or more specimens and 45% had not returned any specimens.[7] They reported that 58% of patients did not return due to inconvenience, 38% embarrassment, 29% confidence in sterility, 17% forgot and 4% were afraid of repeat surgery.[7] There are many other theoretic reasons why men do not return for semen analysis, such as a fear of results and that the semen analyses will be lost or mishandled.[3] Many patients are also not aware that numerous ejaculations may be required to clear the system of sperm cells. Therefore it is important for the physician to take the time to clearly explain the importance of the postvasectomy follow-up protocol for determining sterility.[3] If this is done, we believe that the patient and the surgeon share the responsibility for determining sterility.

A recent study determined the degree of patient compliance with SSAV for those asked to drop off a semen analysis without an appointment compared to those who were provided with an appointment. The addition of an appointment improves patient compliance with first specimen and almost doubles compliance with recommendations for a second semen specimen. Patients provided with a follow-up appointment were more likely to submit initial semen specimens (84% vs. 65 %, *P*-value = 0.0013). The initial noncompliance rate (those not returning any samples at all) of 16 and 35% is similar to rates of 24-36% reported previously.[8–10] These patients were almost twice as likely to provide two consecutive azoospermic semen specimens (38% vs. 20%, *P*-value = 0.0053). However, noncompliance rates increased to 62 and 80% when based on failure to produce two consecutive azoospermic specimens. In addition, all patients with appointments who had evidence of sperm in their samples at two months provided a specimen at three months whereas only 75% of such patients without an appointment provided additional specimens.[11] However, despite aggressive education efforts and scheduled appointments, only 38% of such patients complied with postvasectomy instructions to provide two consecutive azoospermic semen specimens. Similarly, Maatman *et al* reported a noncompliance rate of 73% when based on failure to produce two consecutive azoospermic specimens one month apart.[3] The poor compliance rates further suggest that insisting on two consecutive azoospermic semen analyses may present an unreasonable goal of follow-up.[4]

Insisting on two consecutive azoospermic semen analyses presents potentially insurmountable barriers to patient compliance. Initial noncompliance rates (those returning no samples) have ranged from 24-40%.[9–11] However, noncompliance rates increased to 73-79% when based on failure to produce two consecutive azoospermic SSAVs one month apart.[3,4] Thus, it appears likely that insistence upon two semen analyses may be made in hopes of protecting oneself medico-legally, but is of very limited benefit in assuring sterility.

## THE APPROPRIATE ENDPOINT OF VASECTOMY (RARE NONMOTILE SPERM OR AZOOSPERMIA)

An azoospermic SSAV serves only to confirm division of both vas deferens and does not guarantee that the patient will not develop subsequent re-canalization of the vas deferens. Two consecutive azoospermic SSAVs do not guarantee sterility.[12,13] Studies show that the incidence of the transient re-appearance of sperm after vasectomy is 0.8-2.4%.[14–16]

The persistence of nonmotile sperm after vasectomy is a well-known phenomenon. Even when men had rare nonmotile sperm on their initial (eight-week) semen analysis, we found that at 6-11 months after vasectomy and after submitting an additional one to eight samples, all of these men had azoospermia.[4] De Kniff *et al* reported that 96% of men with RNMS eventually became azoospermic, with a mean (range) follow-up of six (3-21) months and concluded that it was safe to give clearance to patients with RNMS.[5] However, they performed a second vasectomy in the remaining 4% of men with RNMS.

The true failure rate and the recommended follow-up for patients with RNMS has not been established, largely because many of these men are lost to follow-up. The observed failure rate associated with RNMS is reportedly low and several authors have suggested that the finding of RNMS is not an indication for additional testing.[1,6,17] Davies *et al* reported no pregnancies when clearance was given to their 151 patients with RNMS in the SSAV.[4] Chawla *et al* reported a 1% failure rate associated with RNMS, which is only marginally greater than the reported 1 in 2000 late failure rates.[15,18]

Although some reports have indicated that centrifugation of the specimen increases the ability to detect low levels of sperm cells, we do not use centrifugation as a means to confirm azoospermia unless paternity is in question. Despite the possibility that this would identify sperm that are not found in uncentrifuged concurrent semen analyses, we failed to identify a single case where it did so in over 50 patients (unpublished). In addition, examining uncentrifuged semen is the approach used by most urologists.

## EARLY RECANALIZATION AND VASECTOMY FAILURE

Vasectomy failure must be distinguished from postvasectomy recanalization. Late recanalization is usually detected by an unexpected pregnancy. The situation is not as clear with early recanalization which is suspected by the presence of motile sperm at the time of the first sperm count after vasectomy. Previous studies have suggested that the presence of motile sperm after three weeks most probably indicates that a spontaneous recanalization had occurred.[19,20] However, early recanalization does not necessarily imply that the vasectomy has failed, it is believed that most recanalizations eventually close or scar down.[21] Nevertheless, early recanalization is a significant source of burden and anxiety for the patient.

In terms of vasectomy failure, one analysis made of 26 unsuccessful operations from 2,197 vasectomies demonstrated that the most important factor accounting for failure rates was the length of vas excised. They reported that at least 15 mm of vas should be excised to maximize the success of the procedure. Excised vas segments less than 15 mm had up to a 25-fold greater incidence of failure.[22] A recently published article, however found that in their cohort of vasectomized men in whom 5- to 20-mm vas segments were routinely removed during vasectomy, the risk of recanalization was not significantly associated with shorter segments excised.[23] Some of the less well documented cases of late failure have occurred up to 10 years after vasectomy. In the absence of any long-term follow-up studies with regular analyses of semen, it must be assumed that restoration of fertility may occur at any time. However, such events are considered rare; in one study of 14047 men, six wives of men who had undergone vasectomy and in whom zero sperm counts had been recorded after surgery, became pregnant.[24] Of interest, only two of these six men had obvious sperm granulomas. However, the etiologic role of sperm granuloma in the development of recanlization remains hypothetical.

## CONCLUSION

The global increase in the acceptance of vasectomy as the most effective means of male family planning has naturally stimulated intense interest in the consequences of this procedure. Prior to the procedure, information regarding the patient's age, reason for vasectomy and relationship status should be evaluated to determine whether vasectomy is an appropriate option for the patient. In addition, it is important to discuss with the patient alternative methods of contraception, risks of the procedure, which include failure and possible complications such as bleeding and infection. Men should also be encouraged to discuss this decision with their partner.

Vasectomy is one of the most reliable and cost-effective permanent methods of contraception and almost 100 million men worldwide have relied on it for family planning. However, despite its popularity, certain issues pertaining to the procedure remain unresolved. Debate continues over the relative merits of the various techniques of isolating and sealing the vasal ends. Postoperative complication rates remain minimal regardless of the technique used and no single strategy attempting to maximize patient compliance with postoperative semen analysis has enjoyed unmitigated success. Long-term consequences, other than regret, are rare.
